# The Association of Aquaporins with MAPK Signaling Pathway Unveils Potential Prognostic Biomarkers for Pancreatic Cancer: A Transcriptomics Approach

**DOI:** 10.3390/biom15040488

**Published:** 2025-03-26

**Authors:** Inês V. da Silva, Paula A. Lopes, Elisabete Fonseca, Emanuel Vigia, Jorge Paulino, Graça Soveral

**Affiliations:** 1 Research Institute for Medicines (iMed.ULisboa), Faculdade de Farmácia, Universidade de Lisboa, 1649-003 Lisbon, Portugal; 2 Department of Pharmaceutical Sciences and Medicines, Faculdade de Farmácia, Universidade de Lisboa, 1649-003 Lisbon, Portugal; 3 Centro de Investigação Interdisciplinar em Sanidade Animal (CIISA), Faculdade de Medicina Veterinária, Universidade de Lisboa, 1300-477 Lisbon, Portugal; 4 Laboratório Associado para Ciência Animal e Veterinária (AL4AnimalS), Faculdade de Medicina Veterinária, Universidade de Lisboa, 1300-477 Lisbon, Portugal; 5 Hepatobiliopancreatic and Transplantation Center, Hospital de Curry Cabral (CHULC), 1050-099 Lisbon, Portugal; 6 NOVA Medical School, Universidade NOVA de Lisboa, 1169-056 Lisbon, Portugal; 7 Hospital da Luz, 1500-650 Lisbon, Portugal

**Keywords:** aquaporins, epithelial–mesenchymal transition, MAPK/ERK signaling pathway, transcriptomics, pancreatic cancer

## Abstract

Pancreatic cancer is one of the most lethal and challenging malignancies. Its severity is primarily linked to the constitutively activated mitogen-activated protein kinase (MAPK)/extracellular signal-regulated kinase (ERK) pathway. Aquaporins (AQPs) are frequently overexpressed in pancreatic cancer, playing crucial roles in cell signaling, and consequently promoting cell migration, proliferation, and invasion. Here, we investigate the transcriptomics of key players in epithelial–mesenchymal transition (EMT) and the MAPK/ERK signaling pathway in pancreatic cancer tissues, correlating them with tumor AQP expression to highlight their potential as diagnostic or prognostic tools. The transcriptomics analysis was conducted in 24 paired pancreatic tumors and adjacent healthy tissues, and analyses were performed considering the patients’ age and gender, as well as tumor invasiveness and aggressiveness. Our results revealed strong positive Pearson correlation coefficients between *AQP3* and *c-Jun*, and between *AQP5* and *CDH1/EGFR* in pancreatic tumors but not in healthy tissues, with posterior in vitro confirmation in pancreatic cancer BxPC3 cells, suggesting a shift in the regulatory mechanisms of gene expression that certainly affect the physiology of the tissue, influencing cancer initiation and progression. This study underscores the interplay between AQPs and cancer signaling pathways, opening new avenues for defining novel clinical biomarkers and improving the early detection of pancreatic cancer.

## 1. Introduction

Pancreatic cancer is a leading cause of cancer-related deaths worldwide due to its aggressive nature and late-stage diagnosis. According to recent data, in 2020, an estimated 466,000 people died from pancreatic cancer worldwide, and this number is expected to rise as the global burden of cancer grows [1]. Pancreatic cancer accounts for about 4.5% of all cancer deaths, making it one of the deadliest forms of cancer, with a five-year survival rate of approximately 13% globally [2]. The incidence and mortality rates are higher in males compared to females [1]. Age, gender, ethnicity, and genetic background are key non-modifiable risk factors associated with pancreatic cancer [3]. The disease is more commonly observed in elderly individuals and is rare in those under the age of 30.

In this respect, early detection and treatment remain critical, as pancreatic cancer often presents nonspecific symptoms and lacks effective screening tests, contributing to its high mortality rate. The only clinically approved diagnostic biomarker for pancreatic cancer is carbohydrate antigen 19-9 (CA 19-9) which presents a sensitivity of 70–92% and a specificity of 68–92% [4]. The lack of sensitivity of the CA 19-9 test for early detection, combined with the high number of false positive and false negative results, limits its accuracy and usefulness [5]. The current standard-of-care options for pancreatic cancer include surgery, chemotherapy (e.g., FOLFIRINOX, gemcitabine/nab-paclitaxel), targeted therapy, and palliative care. While surgery offers a chance for cure, most patients present unresectable or metastatic disease, leaving chemotherapy as the mainstay of treatment [6]. However, chemotherapy is often associated with limited efficacy due to drug resistance, and only a subset of patients benefit from current targeted therapies, highlighting the need for novel options to improve therapeutic outcomes. Novel and challenging approaches involve targeting cancer stem cells which induce tumor growth, resistance to therapy, and metastasis, and the tumor microenvironment, whose dense stromal matrix protects cancer cells from efficient treatments [7]. Clarifying the network of proteins that interact with the major signaling pathways stands out as a promising approach to unveil novel prognostic biomarkers for human pancreatic cancer.

Pancreatic cancer is driven by alterations in several key signaling pathways that regulate cell growth, survival, invasion, and the tumor microenvironment. KRAS stands as the most commonly mutated gene in pancreatic ductal adenocarcinoma (PDAC), found in over 90% of cases. The most common KRAS mutations in PDAC are G12D (44%), G12V (34%), and G12R (20%). These mutations are not suitable for treatment with KRAS G12C-specific cysteine-reactive inhibitors, such as Sotorasib and Adagrasib, which have demonstrated clinical efficacy in lung cancer. While KRAS G12C-mutant pancreatic cancer has been treated with Sotorasib, this mutation is relatively rare, occurring in only 2–3% of PDAC cases [8]. Mutations in KRAS lead to continuous activation of downstream pathways, such as the MAPK/ERK and PI3K/AKT pathways, driving uncontrolled cell proliferation and survival [9]. The key players in MAPK/ERK and PI3K/AKT pathways represent potential targets to prevent this oncogenic signaling cascade.

Aquaporins (AQPs) are integral membrane proteins that facilitate the transport of water and small solutes, such as glycerol and H_2_O_2_, across cell membranes [10,11,12]. Beyond their traditional role in cellular homeostasis, AQPs are increasingly recognized for their involvement in cancer biology, including pancreatic cancer [11,13,14,15,16,17]. AQPs’ implication in pancreatic cancer lies in their contribution to cell morphological changes, membrane biomechanical properties, cell–cell adhesion, tumor growth, invasion, metastasis, and interaction with pivotal signaling pathways [18,19,20,21,22,23]. In particular, AQP1, AQP3, and AQP5 enhance the motility and invasiveness of pancreatic cancer by facilitating actin cytoskeleton remodeling, enabling cancer cells to migrate efficiently through tissue barriers [22,24,25]. A few studies have suggested that AQP5 is involved in promoting epithelial-to-mesenchymal transition (EMT), a key step in cancer metastasis [19,26], and that transforming growth factor-β (TGF-β)-induced EMT modulates AQP3 expression supporting tumor invasion and the establishment of metastatic niches [27]. Additionally, KRAS mutations, prevalent in pancreatic cancer, might upregulate AQPs, indirectly contributing to tumor progression through enhanced cell proliferation and survival mechanisms [28,29,30,31]. AQP3 has been linked to the activation of PI3K/AKT signaling, which promotes cell growth, resistance to apoptosis, and chemoresistance [32]. AQP1 is implicated in angiogenesis, helping to supply growing tumors with nutrients and oxygen [33,34]. AQP3 also facilitates the transport of glycerol, supporting metabolic flexibility and energy production in cancer cells under hypoxic or nutrient-deprived conditions, common in pancreatic tumors [17]. Several studies have shown that AQP blockage or silencing can suppress tumor growth, angiogenesis, and metastasis [22,24,35,36]. Conversely, AQP9, the most representative AQP in the liver, is downregulated in hepatocellular carcinoma and its overexpression suppresses hepatoma cell invasion via the epithelial–mesenchymal transition inhibition [37]. Moreover, AQPs, particularly AQP5, have been implicated in promoting tumorigenesis through the activation of intracellular signaling pathways, including the MAPK/ERK pathway [38,39,40]. Recently, we reported the modulation of the most cancer-associated AQPs—AQP1, AQP3, AQP5, and AQP9—in paired pancreatic tumors and adjacent healthy tissues from a pancreatic cancer cohort [41]. This study highlighted the downregulation of AQP1 in tumor tissues compared to healthy tissues. Additionally, AQP3 was particularly overexpressed in low-grade invasive tumors. Interestingly, most of the positive Pearson correlation coefficients found among all AQPs in healthy tissues were lost in tumor tissues, suggesting a disrupted regulation of the AQP genes network in pancreatic cancer [41].

Given the established role of the MAPK pathway in pancreatic cancer progression and its potential as a therapeutic target, our study aims to investigate whether AQPs are associated with MAPK activity. The fact that AQPs are key players in various cancer processes and these events are correlated with KRAS continuous activation, prompted us to investigate the relationship between AQPs and the MAPK/ERK pathway. This correlation could provide insights into their biological significance and identify novel prognostic biomarkers.

In the present study, the gene expression of key players in EMT—*CDH1* and *VIM*—and the MAPK/ERK pathway—*EGFR*, *ERK1*, *ERK2*, *c-Jun*, and *c-Fos*—was assessed in paired healthy and neoplastic tissues of the same pancreatic cancer cohort. The modulation of these genes’ expression was analyzed, according to the age range and gender of the patients and the grade of tumor invasiveness and aggressiveness, and was further correlated with the gene expression of *AQP1*, *AQP3*, *AQP5*, and *AQP9*, previously reported [41]. The positive correlations found were further validated in vitro in human pancreatic cancer cultured cells, aiming to understand the complex network of AQPs and key players in EMT and MAPK/ERK signaling pathways involved in the settings of pancreatic cancer.

## 2. Materials and Methods

### 2.1. Ethical Approval

All procedures involving patients were conducted following the ethical standards established by the local research committees and stated in the original Declaration of Helsinki (1964), and subsequent amendments in 2000 [42]. Paired healthy and neoplastic tissue samples were collected from 24 patients with pancreatic cancer who underwent surgical procedures. Written informed consent was obtained from all patients before the samples’ collection. The ethical approval for this study was obtained from the Ethics Committee of the Centro Hospitalar de Lisboa Central (CHULC) (reference INV-106, 2021).

### 2.2. Pancreatic Cancer Cohort Characterization

Patients were classified using the pathological tumor/node/metastasis (pTNM) system, based on the guidelines outlined in the 7th edition of the American Joint Committee on Cancer (AJCC). The clinicopathological characterization of the cohort is listed in Table 1. The samples were obtained from 24 patients with pancreatic cancer, 13 male and 11 female patients, aged 64.3 ± 9.5 years old at the time of surgery, and were classified according to the aggressiveness and invasiveness grades of pancreatic cancer by both surgeons and pathologists.

Tumor aggressiveness was evaluated based on the histological type and grade, with classification ranging from 1 to 4. Accordingly, the samples were divided into two groups: a low-grade aggressive group (patients with an aggressiveness grade ≤2) and a high-grade aggressive group (patients with an aggressiveness grade ≥3). The invasiveness grade was determined based on the extent of local tumor invasion and lymph node involvement, also classified from 1 to 4. Based on this evaluation, the samples were categorized into a low-grade invasive group (invasiveness grade = 1) and a high-grade invasive group (invasiveness grade ≥2).

### 2.3. Cell Culture

The BxPC-3 cell line was purchased in ATCC (catalog no. CRL-1687). Cells were grown in RPMI 1640 medium (Gibco, Thermo Fisher Scientific, Waltham, MA, USA) supplemented with 10% heat-inactivated Fetal Bovine Serum (FBS; Gibco, Thermo Fisher Scientific, Waltham, MA, USA) at 37 °C, 5% CO_2_.

### 2.4. Cell Transfection

The knockdown of AQP3 and AQP5 was performed in BxPC3 cells using small interfering RNA (siRNA)-containing expression vectors (ID: s1521 and ID: s1527, respectively, Ambion, Thermo Fisher Scientific Waltham, MA, USA) combined with Lipofectamine RNAiMAX Reagent (Invitrogen, Waltham, MA, USA). A control of silencing (Silencer Negative Control siRNA #1, Ambion, Thermo Fisher Scientific, Waltham, MA, USA) was performed in parallel. For transfections, cells were seeded with an inoculum of 2 × 104 cells/cm^2^ in 12-well plates (Thermo Fisher Scientific, Waltham, MA, USA). Transfections were validated by qPCR and functional assays as described before [22,24]. Cells were used for experiments 48 h post-transfection. The experimental groups were established as follows: BxPC-3 cells transfected with negative control (siCTL), BxPC-3 cells transfected with AQP3 siRNA (siAQP3), and BxPC-3 cells transfected with AQP5 siRNA (siAQP5).

### 2.5. RNA Extraction

For RNA isolation, healthy and neoplastic tissue samples were homogenized in TRIzol (Invitrogen, Waltham, MA, USA), using a TissueRuptor II (Qiagen, Hilden, Germany). RNA was extracted according to the manufacturer’s protocol (Invitrogen, Waltham, MA, USA). The quantity and integrity of the mRNA were assessed using a Nanodrop 2000c spectrophotometer (Thermo Fisher Scientific, Waltham, MA, USA), and only samples with the required quality (260/280 and 260/230 ratio values of around 2) were used in the following step.

### 2.6. cDNA Synthesis

The cDNA was synthesized from 500 ng RNA and performed using the NZY First-Strand cDNA Synthesis Kit, according to the manufacturer’s protocol (NZYTech, Lisbon, Portugal) in a CFX96 Real-Time System C1000 (Bio-Rad Laboratories, Hercules, CA, USA), as previously described [36].

### 2.7. Quantitative Polymerase Chain Reaction (qPCR)

The gene expression of *CDH1*, *VIM*, *EGFR*, *ERK1*, *ERK2*, *c-Jun*, and *c-Fos* was studied using the Expert SYBR Green qPCR Master Mix (GRISP, Lisbon, Portugal). *HPRT1* was used as the housekeeping gene. Primers are listed in Table 2. Primer efficiency was validated using a 10-fold serial dilution of the cDNA template, spanning 3 dilution points. The resulting standard curve was generated by plotting the Ct values against the logarithm of the template concentration. Amplification efficiency was calculated from the slope of the standard curve using the formula E = (10^(−1/slope)^ − 1) × 100, ensuring efficiency values were within the acceptable range of 90–110%. The gene expression of aquaporins was performed using the TaqMan Universal PCR Master Mix (Applied Biosystems, ThermoFisher Scientific, Waltham, MA, USA), and the following specific TaqMan pre-designed gene expression primers: *AQP0* (Hs0085175_m1), *AQP1* (Hs01028916_m1), *AQP2* (Hs00166640_m1), *AQP3* (Hs01105469_g1), *AQP4* (Hs00242342_m1), *AQP5* (Hs00387048_m1), *AQP6* (Hs00155808_m1), *AQP7* (Hs00357359_m1), *AQP8* (Hs01086280_g1), *AQP9* (Hs00175573_m1), *AQP10* (Hs00369738_m1), *AQP11* (Hs005426181_m1), and *AQP12* (Hs01651303_m1) (Applied Biosystems, Thermo Fisher Scientific, Waltham, MA, USA). *β-ACT* (Hs99999903_m1) and *HPRT-1* were used as housekeeping genes in cell cultures and tissues. qPCR reactions were carried out using a CFX96 Real-Time System C1000 (Bio-Rad Laboratories, Hercules, CA, USA). The relative quantification of gene expression was determined using a modified Livak method [43]. The gene expression was normalized to the housekeeping gene. This procedure was performed in triplicate.

### 2.8. Statistical Analysis

Statistics were performed by applying the generalized linear mixed (GLM) model of Statistical Analysis System (SAS) software, version 9.4 [44]. After normality was tested by the Kolmogorov–Smirnov test and variance homogeneity was tested by Levene’s test, significant multiple comparisons were performed using the PDIFF option adjusted with Tukey–Kramer to determine statistical differences among healthy and pancreatic cancer tissues. Pearson correlation coefficients were calculated using the Proc CORR procedure of SAS to find linear relationships among genes’ transcriptional profiles. Data were expressed as the mean and standard error of the mean (SEM). *p* < 0.05 was chosen as the cut-off for statistical significance.

### 2.9. TNMplot Database Analysis

TNMplot web tool (TNMplot.com) was used to validate Pearson correlations between gene pairs in GeneChip data. Gene expression data from tumor samples were analyzed to confirm the correlation trends observed in our dataset. Pearson correlation coefficients were calculated within TNMplot, and the results were compared to our initial findings to ensure consistency and robustness. This approach provided independent validation of gene-gene correlations, reinforcing the reliability of our analyses in the context of cancer progression.

## 3. Results

### 3.1. Association of Tumor and Age Range or Gender with the EMT Process

**Figure 1:**
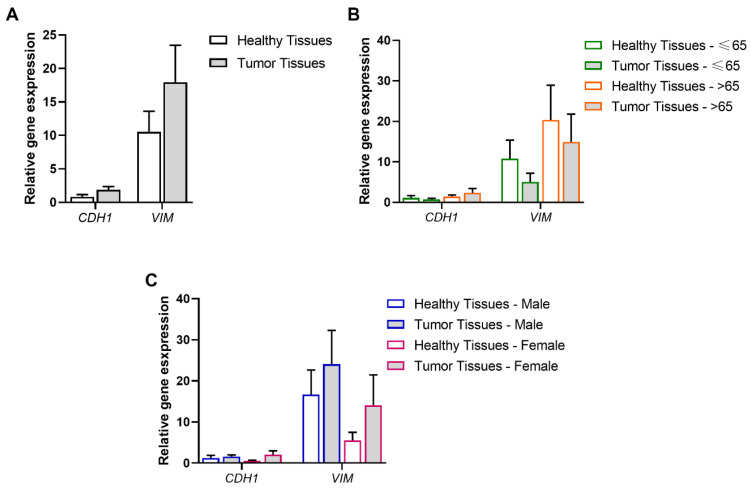
Association of pancreatic tissue type and age range or gender with the EMT process. (**A**) Modulation of *CDH1*, the marker of cell polarity, and *VIM*, the marker of mesenchymal cells, in pancreatic tumor tissues (*n* = 24) compared to healthy tissues (*n* = 24). (**B**) Modulation of *CDH1* and *VIM* in healthy and pancreatic tumor tissues, according to patients’ age range (≤65 or >65 years old) (healthy ≤ 65 (*n* = 14), tumor ≤65 (*n* = 14), healthy > 65 (*n* = 10), tumor > 65 (*n* = 10)). (**C**) Modulation of *CDH1* and *VIM* in healthy and pancreatic tumor tissues, according to patients’ gender (male or female) (healthy male (*n* = 13), tumor male (*n* = 13), healthy female (*n* = 11), tumor female (*n* = 11)). Values are mean ± SEM.

*CDH1* and *VIM* transcript expression were analyzed by qPCR, according to different variables to ascertain associations with patients’ specific features and predisposition to develop pancreatic cancer. The analyses were carried out as follows: healthy (*n* = 24) vs. pancreatic tumor (*n* = 24) tissues (Figure 1A), healthy vs. pancreatic tumor tissues, each sub-grouped according to the patients’ age (≤65 or >65 years old; healthy ≤65, *n* = 14; tumor ≤65, *n* = 14; healthy >65, *n* = 10; tumor >65, *n* = 10) (Figure 1B); and healthy vs. pancreatic tumor tissues, each sub-grouped according to the patients’ gender (male or female; healthy male, *n* = 13; tumor male, *n* = 13; healthy female, *n* = 11; tumor female, *n* = 11) (Figure 1C).

*CDH1*, the epithelial cell differentiation marker, was expressed in both pancreatic tissue types; however, no significant differences were observed when comparing healthy vs. tumor tissues (*CDH1*: *p* > 0.05). Similarly, *VIM*, the mesenchymal cell marker, was also detected in healthy and tumor tissues but no differences were observed when comparing both conditions (*VIM*: *p* > 0.05) (Figure 1A).

When the healthy and tumor groups were reorganized according to the patients’ age range (≤65 or >65 years old, Figure 1B) and the patients’ gender (male or female, Figure 1C), no statistically significant differential expression of *CDH1* and *VIM* transcript levels were observed (*p* > 0.05). These data show that, in this pancreatic cancer cohort, *CDH1* and *VIM* relative expression is not age- or gender-dependent.

### 3.2. Association of Tumor Aggressiveness and Invasiveness with the EMT Process

**Figure 2:**
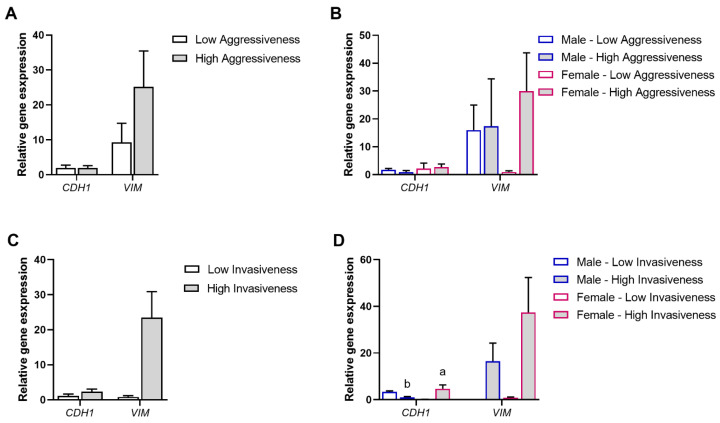
Association of pancreatic tumor tissue aggressiveness or invasiveness and gender with the EMT process. (**A**) Modulation of *CDH1*, a marker of cell polarity, and *VIM*, a marker of mesenchymal cells, in pancreatic tumor tissues, according to their aggressiveness grade (low (*n* = 14) or high (*n* = 10)). (**B**) Modulation of *CDH1* and *VIM* in pancreatic tumor tissues, according to the tumor aggressiveness (low or high) and the patients’ gender (male or female) (low aggressiveness male (*n* = 9), high aggressiveness male (*n* = 4), low aggressiveness female (*n* = 5), high aggressiveness female (*n* = 6)). (**C**) Modulation of *CDH1* and *VIM* in pancreatic tumor tissues, according to their invasiveness grade (low (*n* = 7) or high (*n* = 17)). (**D**) Modulation of *CDH1* and *VIM* in pancreatic tumor tissues, according to the tumor invasiveness (low or high) and the patients’ gender (male or female) (low invasiveness male, (*n* = 2), high invasiveness male (*n* = 11), low invasiveness female (*n* = 5), high invasiveness female (*n* = 6)). Values are mean ± SEM. Mean values with unlike letters were significantly different (Tukey’s post hoc, *p* < 0.05).

EMT enables the detachment of cancer cells from primary tumors, allowing the invasion of surrounding tissues and migration to distant sites, consequently influencing cancer metastasis [45]. Transcript expression levels of *CDH1* and *VIM* were also analyzed according to the aggressiveness and invasiveness grade of tumors to ascertain associations with such pancreatic cancer features. The analysis was performed according to the following variables: tumors with low (*n* = 14) vs. high aggressiveness (*n* = 10) (Figure 2A), tumors with low vs. high aggressiveness, each sub-grouped according to the patients’ gender (male or female; low aggressiveness male, *n* = 9; high aggressiveness male, *n* = 4; low aggressiveness female, *n* = 5; high aggressiveness female, *n* = 6) (Figure 2B), tumors with low (*n* = 7) vs. high invasiveness (*n* = 17) (Figure 2C), tumors with low vs. high invasiveness, each sub-grouped according to the patients’ gender (male or female; low invasiveness male, *n* = 2; high invasiveness male, *n* = 11; low invasiveness female, *n* = 5; high invasiveness female, *n* = 6) (Figure 2D).

*CDH1* and *VIM* expression levels were found to be similar in low- and high-aggressiveness grade tumors (*p* > 0.05) (Figure 2A). Moreover, when low- and high-aggressiveness grade tumors were reorganized according to the patients’ gender (male or female), no significant differences were observed in *CDH1* and *VIM* transcript levels (*p* > 0.05) (Figure 2B).

However, when considering invasiveness, *CDH1* and *VIM* relative expression levels showed increased mean values in high invasiveness compared to low invasiveness tumors, although not reaching statistical significance (*p* > 0.05) (Figure 2C). While *VIM* expression was not statistically different between genders (*p* > 0.05), a tendency for higher values in high-invasiveness grade tumors from female patients was observed (*p* = 0.061). Moreover, *CDH1* relative expression was significantly higher in high-invasiveness grade tumors from female patients than from male patients (*p* < 0.05). *VIM* expression was not statistically different between invasiveness grades within the same gender (*p* > 0.05) (Figure 2D).

### 3.3. Association of Tumor and Age Range or Gender with the MAPK/ERK Signaling Pathway

**Figure 3:**
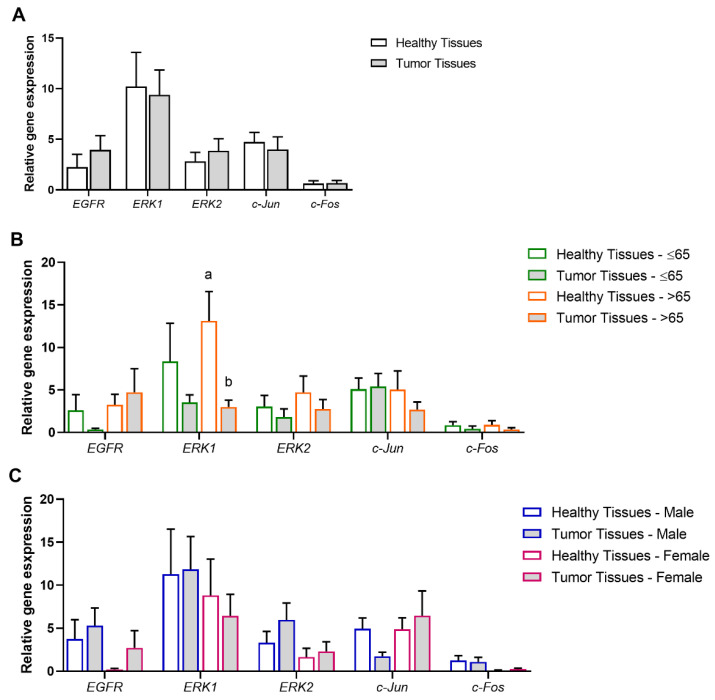
Association of pancreatic tissue type and age range or gender with the MAPK/ERK signaling pathway. (**A**) Modulation of *EGFR*, a membrane receptor, *ERK1* and *ERK2*, two MAPK kinases, and *c-Jun* and *c-Fos*, two transcription factors, in pancreatic tumor tissues (*n* = 24) compared to healthy tissues (*n* = 24). (**B**) Modulation of *EGFR*, *ERK1*, *ERK 2*, *c-Jun*, and *c-Fos* in healthy and pancreatic tumor tissues, according to the patients’ age range (≤65 or >65 years old) (healthy ≤65 (*n* = 14), tumor ≤65 (*n* = 14), healthy >65 (*n* = 10) tumor >65 (*n* = 10)). (**C**) Modulation of *EGFR*, *ERK1*, *ERK 2*, *c-Jun*, and *c-Fos* in healthy and pancreatic tumor tissues according to the patients’ gender (male or female) (healthy male (*n* = 13), tumor male (*n* = 13), healthy female (*n* = 11) tumor female (*n* = 11)). Values are mean ± SEM. Mean values with unlike letters are significantly different (Tukey’s post hoc, *p* < 0.05).

The expression of key genes for MAPK/ERK signaling, *EGFR*, *ERK1*, *ERK2*, *c-Jun*, and *c-Fos*, was analyzed according to different variables to investigate associations between patients’ specific features and the predisposition to develop pancreatic cancer. The analyses were carried out as follows: healthy (*n* = 24) vs. pancreatic tumor (*n* = 24) tissues (Figure 3A), healthy vs. pancreatic tumor tissues, each sub-grouped according to the patients’ age range (≤65 or >65 years old; healthy ≤ 65, *n* = 14; tumor ≤ 65, *n* = 14; healthy > 65, *n* = 10; tumor > 65, *n* = 10) (Figure 3B), and healthy vs. pancreatic tumor tissues, each sub-grouped according to the patients’ gender (male or female; healthy male, *n* = 13; tumor male, *n* = 13; healthy female, *n* = 11; tumor female, *n* = 11) (Figure 3C).

*EGFR*, the membrane receptor, *ERK1* and *ERK2*, two MAPK kinases, and *c-Jun* and *c-Fos*, two transcription factors, were detected in both healthy and pancreatic tumor tissues and no differences in relative expression were observed when comparing conditions (*p* > 0.05) (Figure 3A).

Then, healthy and tumor groups were reorganized according to the patient’s age range (≤65 or >65 years old). In healthy tissues, *EGFR*, *ERK1*, *ERK2*, *c-Jun*, and *c-Fos* relative expression were not age-dependent (*p* > 0.05). In turn, in pancreatic tumor tissues, lower *ERK1* transcript levels were detected in tumor tissues of patients >65 years old (*p* < 0.05) than in healthy tissues within the same age group. No other differences in gene expression were observed when comparing the same age group and different pancreatic tissue type or between groups with different age ranges and the same pancreatic tissue type (*p* > 0.05) (Figure 3B).

When healthy and tumor groups were reorganized according to the patient’s gender (male or female), no significant differences were observed in *CDH1* and *VIM* transcript levels (*p* > 0.05). Therefore, the present data suggests that for this pancreatic cancer cohort, *EGFR*, *ERK1*, *ERK2*, *c-Jun*, and *c-Fos* transcript levels are not gender-dependent (Figure 3C).

### 3.4. Association of Tumor Aggressiveness and Invasiveness with the MAPK/ERK Signaling Pathway

**Figure 4:**
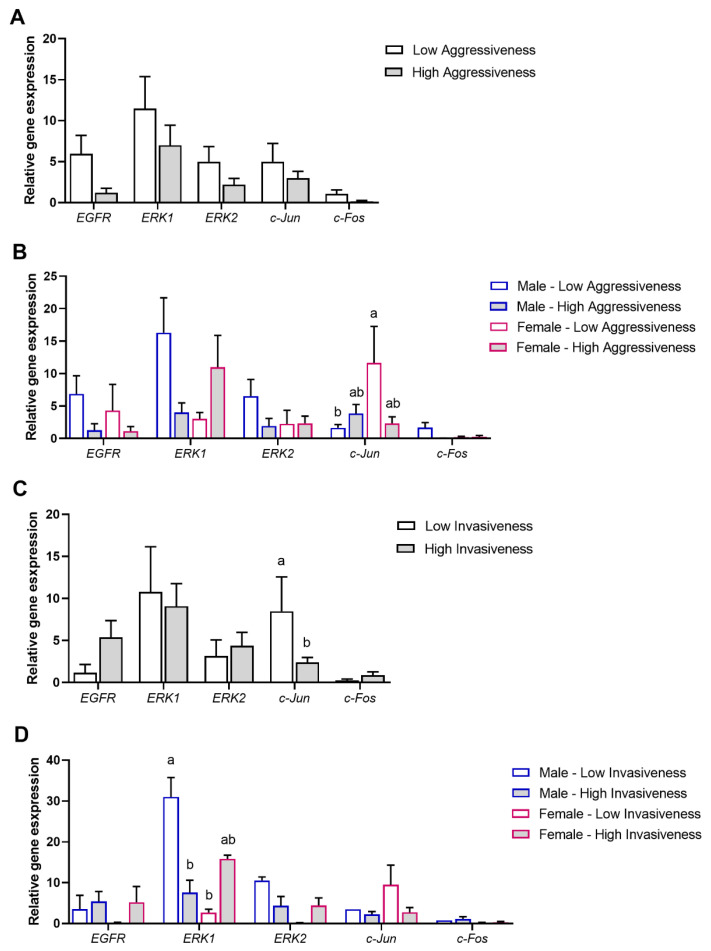
Association of pancreatic tumor tissue aggressiveness or invasiveness and gender with the MAPK/ERK signaling pathway. (**A**) Modulation of *EGFR*, a membrane receptor, *ERK1* and *ERK2*, two MAPK kinases, and *c-Jun* and *c-Fos*, two transcription factors, in pancreatic tumor tissues, according to their aggressiveness grade (low (*n* = 14) or high (*n* = 10)). (**B**) Modulation of *EGFR*, *ERK1*, *ERK2*, *c-Jun*, and *c-Fos* in pancreatic tumor tissues, according to the tumor aggressiveness (low or high) and to the patients’ gender (male or female) (low aggressiveness male (*n* = 9), high aggressiveness male (*n* = 4), low aggressiveness female (*n* = 5), high aggressiveness female (*n* = 6)). (**C**) Modulation of *EGFR*, *ERK1*, *ERK2*, *c-Jun*, and *c-Fos* in pancreatic tumor tissues, according to their invasiveness grade (low (*n* = 7) or high (*n* = 17)). (**D**) Modulation of *EGFR*, *ERK1*, *ERK2*, *c-Jun*, and *c-Fos* in pancreatic tumor tissues, according to the tumor invasiveness (low or high) and to the patients’ gender (male or female) (low invasiveness male (*n* = 2), high invasiveness male (*n* = 11), low invasiveness female (*n* = 5), high invasiveness female (*n* = 6)). Values are mean ± SEM. Mean values with unlike letters are significantly different (Tukey’s post hoc, *p* < 0.05).

The relative gene expression of *EGFR*, *ERK1*, *ERK 2*, *c-Jun*, and *c-Fos* was also evaluated according to the aggressiveness and invasiveness grade of the pancreatic tumors. The analyses were performed, according to the following variables: tumors with low (LA; *n* = 14) vs. high aggressiveness (HA; *n* = 10) (Figure 4A), tumors with low vs. high aggressiveness, each sub-grouped according to the patients’ gender (male or female; low aggressiveness male, *n* = 9; high aggressiveness male, *n* = 4; low aggressiveness female, *n* = 5; high aggressiveness female, *n* = 6) (Figure 4B), tumors with low (LI; *n* = 7) vs. high invasiveness (HI; *n* = 17) (Figure 4C), tumors with low vs. high invasiveness, each sub-grouped according to the patients’ gender (male or female; low invasiveness male, *n* = 2; high invasiveness male, *n* = 11; low invasiveness female, *n* = 5; high invasiveness female, *n* = 6) (Figure 4D).

Our results show that *EGFR*, *ERK1*, *ERK 2*, *c-Jun*, and *c-Fos* transcript levels were statistically similar in low- and high-aggressiveness grade tumors (*p* > 0.05) (Figure 4A). When low- and high-aggressiveness grade tumors were reorganized according to the patient’s gender (male or female), significantly higher c-Jun transcript levels were observed in female patients with lower-aggressiveness grade tumors than in male patients with lower-aggressiveness grade tumors (*p* < 0.05). No other significant difference was observed for other gene expression (*p* > 0.05) (Figure 4B).

Regarding invasiveness, c-Jun relative expression was higher in low-invasiveness grade tumors than in high-invasiveness grade tumors (*p* < 0.05). The expression level of *EGFR*, *ERK1*, *ERK 2*, and *c-Fos* was similar in the two tested conditions (Figure 4C). Curiously, after reorganizing low- and high-invasiveness grade tumors according to the patient’s gender (male or female), higher levels of *ERK1* in male patients with low-invasiveness grade tumors were observed compared to high-invasiveness grade tumors (*p* < 0.05). Higher levels of *ERK1* in male patients with low-invasiveness grade tumors were observed compared to female patients with low-invasiveness grade tumors (*p* < 0.05). No other statistical differences were observed between different invasiveness grades and genders (*p* > 0.05) (Figure 4D).

### 3.5. Pearson Correlation Coefficients Between AQPs, EMT, and MAPK/ERK Signaling Pathways

In a previous publication, we investigated the prognostic value of AQP transcripts in the same pancreatic cancer cohort and discussed the correlations found among various AQP isoforms (*AQP1*, *AQP3*, *AQP5*, and *AQP9*) [41]. Herein, AQP transcripts were correlated with markers of EMT and MAPK/ERK signaling pathways.

Table 3 and Table 4 depict Pearson correlation coefficients between transcript levels of *AQP*s, *CDH1*, *VIM*, *EGFR*, *ERK1*, *ERK2*, *c-Jun*, and *c-Fos* in healthy (Table 3), and pancreatic tumor (Table 4) tissues. All the correlations were found to be positive (high correlation, r > 0.7; moderate correlation, 0.7 ≥ r ≥ 0.3; low correlation, r < 0.3) [46].

In healthy tissues, *CDH1* was correlated with *VIM* (r = 0.487, *p* < 0.05) and *c-Fos* (r = 0.892, *p* < 0.05). *VIM* was correlated with *ERK1* (r = 0.505, *p* < 0.05), *ERK2* (r = 0.707, *p* < 0.05), and *c-Fos* (r = 0.742, *p* < 0.05). *EGFR* was correlated with *c-Fos* (r = 0.617, *p* < 0.05). *ERK1* was correlated with *ERK2* (r = 0.842, *p* < 0.05) and *c-Fos* (r = 0.607, *p* < 0.05). *ERK2* was correlated with *c-Fos* (r = 0.567, *p* < 0.05) (Table 3). Interestingly, for healthy tissues, none of the evaluated AQPs were shown to correlate with key players in tumorigenesis (*p* > 0.05).

In pancreatic tumor tissues, *CDH1* was correlated with *EGFR* (r = 0.744, *p* < 0.05) and *ERK1* (r = 0.689, *p* < 0.05). *VIM* was correlated with *ERK1* (r = 0.719, *p* < 0.05). *ERK1* was correlated with *ERK2* (r = 0.796, *p* < 0.05) and c-Fos (r = 0.567, *p* < 0.05). *ERK2* was correlated with *c-Fos* (r = 0.667, *p* < 0.05). Interestingly, *AQP5* was correlated with *CDH1* (r = 0.737, *p* < 0.05) and *EGFR* (r = 0.457, *p* < 0.05). *AQP3* was correlated with *c-Jun* (r = 0.852, *p* < 0.05). *AQP9* was correlated with *c-Jun* (r = 0.952, *p* < 0.05) (Table 4).

To further validate the correlations found, we analyzed publicly available datasets using TNMplot and observed significant correlations between *AQP3* and *c-Jun* (*p* < 0.01), and *AQP5* with *EGFR* (*p* = 0.040) and *CDH1* (*p* < 0.01) in pancreatic cancer. Notably, while our tissue analyses initially suggested a correlation between *AQP9* and *c-Jun*, this was not confirmed in the TNMplot dataset (*p* = 0.32).

### 3.6. Experimental Validation of the Positive Associations Between AQPs and Markers of EMT and MAPK/ERK Signaling Pathways

**Figure 5:**
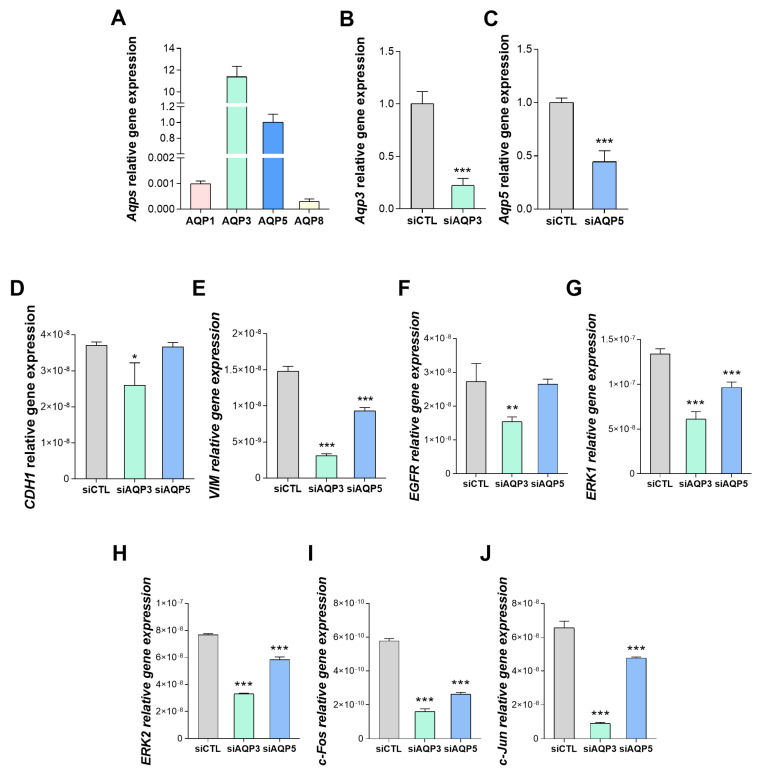
Effect of *AQP3* and *AQP5* silencing on the transcriptional profile of EMT and MAPK/ERK signaling pathways in human pancreatic cancer cells. (**A**) Relative expression levels of AQP isoforms detected in BxPC3 cells. (**B**) *AQP3* and (**C**) *AQP5* relative expression levels in control and siRNA-silenced BxPC3 cells. Relative expression levels of markers of the EMT signaling pathway: (**D**) *CDH1*, and (**E**) *VIM*; and MAPK/ERK signaling pathway (**F**) *EGFR*, (**G**) *ERK1*, (**H**) *ERK2*, (**I**) *c-Fos*, and (**J**) *c-Jun*, in control, siAQP3 and siAQP5 silenced BxPC3 cells. Values are mean ± SEM. *n* = 3 independent experiments. *, *p* < 0.05; **, *p* < 0.01; ***, *p* < 0.001.

To validate the results obtained by the Pearson correlation coefficients, we investigated the impact of AQPs in regulating cellular processes associated with EMT and MAPK/ERK signaling pathways in vitro. For that, we cultured a pancreatic cancer cell line, BxPC3, that was screened for AQP expression, revealing high levels of *AQP3* and *AQP5* expression and much lower levels of *AQP1* and *AQP8* (Figure 5A). Consequently, cells were silenced for *AQP3* (Figure 5B) and *AQP5* expression (Figure 5C) using siRNAs, resulting in a 78% and 65% reduction of relative gene expression levels, respectively. Then, the impact of *AQP3* and *AQP5* knockdown on EMT and MAPK/ERK signaling pathways was assessed by analyzing the relative gene expression of *CDH1*, *VIM*, *EGFR*, *ERK1*, *ERK2*, *c-Fos* and *c-Jun*. *AQP3* knockdown resulted in decreased transcription of all tested marker genes for both EMT and MAPK/ERK signaling pathways (Figure 5D–J). *AQP5* silencing resulted in impaired levels of *VIM*, *ERK1/2*, *c-Fos*, and *c-Jun* (Figure 5D–J).

## 4. Discussion

The incidence of pancreatic cancer is steadily increasing worldwide. Developing innovative and reliable methods for early diagnosis and prognosis is paramount. Identifying novel biomarkers, such as AQPs and their associated signaling pathways, addresses the critical weaknesses of existing markers, like CA19-9, for pancreatic cancer by offering additional options and possibly increased sensitivity, specificity, and prognostic value. Once validated, these biomarkers hold the potential for earlier diagnosis, better treatment stratification, and non-invasive monitoring, ultimately contributing to improved outcomes in pancreatic cancer management.

In the present study, we evaluated the expression of *CDH1*, *VIM*, *EGFR*, *ERK1*, *ERK2*, *c-Jun*, and *c-Fos* in 24 paired healthy and tumor tissues from pancreatic cancer patients who underwent resection surgery. The transcriptomics was correlated with the patients’ age range, gender, and the grades of tumor aggressiveness and invasiveness. Recently, we assessed the expression of tumor-associated AQPs (*AQP1*, *AQP3*, *AQP5*, and *AQP9*) in the same pancreatic cancer cohort, revealing *AQP1* downregulation in tumor tissues, and *AQP3* upregulation in low-invasiveness grade tumors compared to high invasiveness grade pancreatic tumors [41]. Here, we took advantage of the data obtained in previous studies and correlated the transcriptomics to find associations between AQPs gene expression and EMT or MAPK/ERK signaling pathways to highlight their clinical significance in pancreatic cancer diagnostics and prognostics. AQPs, which are commonly found upregulated in a variety of malignancies, such as breast cancer [47,48], cervical cancer [49,50], colon cancer [51,52], lung cancer [53,54], ovarian cancer [55], prostate cancer [56,57], and pancreatic cancer [18,41,58], have been shown to be significantly implicated in tumor recurrence, metastasis and poorer patient prognosis by enhancing cancer cell proliferation, migration, and survival through multiple signaling pathways that remain to be fully understood [14,16,40,59]. To the best of our knowledge, this is the first report on the association of *AQP1*, *AQP3*, *AQP5*, and *AQP9* and EMT/MAPK pathways in a cohort of pancreatic cancer patients.

Here, we demonstrated that female patients with high-invasiveness grade tumors exhibited higher levels of *CDH1* and *VIM* transcripts compared to female patients with low-invasiveness grade tumors. Additionally, female patients with high-invasiveness grade tumors displayed higher *CDH1* transcript levels than male patients. E-cadherin, an epithelial cell marker, and Vimentin, a mesenchymal cell marker, are key players in EMT. During EMT, E-cadherin expression decreases and Vimentin increases, and cells lose their epithelial characteristics and differentiation, regaining mesenchymal cell features, such as enhanced motility and reduced cell–cell adhesiveness [60]. Therefore, EMT plays a crucial contribution to rapid cancer progression, with pancreatic malignancies characterized by early invasion and metastasis [61].

Contrary to expected, *AQP5* was positively correlated with *CDH1* in pancreatic tumor tissues. In fact, several AQPs have been implicated in the EMT process. AQP5 promotes invasiveness [62] and increased AQP5 was associated with impaired E-cadherin, α-catenin (cat), and γ-cat, along with increased fibronectin and Vimentin [19,62]. However, while E-cadherin is widely recognized as a suppressor of tumor progression due to its role in maintaining cell–cell adhesion, recent studies suggest that its expression can be context-dependent and influenced by various factors, including tumor microenvironment, cancer type, and stage of progression. In fact, in some tumor contexts, E-cadherin expression is retained in less invasive tumor regions or during early tumor progression stages somehow associated with the prevention of tumor progression into a more invasive type [63,64]. Moreover, AQP5 has been implicated in pathways that may influence epithelial characteristics and adhesion properties, which could explain the observed correlation under specific conditions. Previously, we showed that AQP5 increases in the early stages of pancreatic ductal adenocarcinoma and decreases in the late stages [19], which correlates with the observations that we report in the present study. Other AQPs, such as AQP1, AQP3, and AQP9, have also been previously implicated in the EMT process. The upregulation of AQP1 and AQP3 promotes the downregulation of E-cadherin and the upregulation of Vimentin, enhancing the mesenchymal transformation [65,66]. EGF-induced AQP3 increase is associated with augmented cell migration, invasion, and metastasis [67,68,69]. Conversely, AQP9 is found downregulated in hepatocellular carcinoma, and its overexpression prevents growth and EMT, thereby reducing hepatic cancer invasion and metastasis [37,70].

Concerning the MAPK/ERK signaling pathway, which is constitutively activated in pancreatic cancer cells, we have shown that pancreatic tumor tissues from patients >65 years old are associated with reduced *ERK1* transcript levels than in healthy tissues within the same age range. Additionally, high levels of *ERK1* were associated with low-invasiveness grade tumors in male patients compared to high-grade tumors. Such high *ERK1* expression was also more prominent in male patients with low-invasiveness grade tumors than in female patients with the same tumor features.

ERK1 and ERK2 as key players in the MAPK/ERK signaling pathway are critically involved in many cellular processes, such as proliferation, and cell survival [71]. MAPK cascades are activated by cell-surface receptors, such as EGFR, via cytoplasmic signaling proteins and consist of signaling transmission partially based on phosphorylation/dephosphorylation of consecutive protein kinases, MAPKKK, MAPKK, and MAPK. ERK1/2 phosphorylation promotes the activation of multiple cytoplasmic and nuclear proteins, such as transcription factors c-Jun and c-Fos, involved in the activation of various biological functions [72]. In this pancreatic cancer cohort, increased levels of *c-Jun* were observed in female patients with lower-aggressiveness grade tumors than in male patients with comparable tumor features. When evaluating invasiveness, higher *c-Jun* expression was observed in low-grade tumors than in high-grade tumors, regardless of the patient’s gender.

Curiously, Pearson correlation coefficients showed an association between *VIM* and *ERK1*, suggesting a regulation linking EMT and MAPK pathway. In fact, the Vimentin–ERK axis is described to regulate the transcription of Slug, a transcription factor that promotes differentiation and migration, and the 3 proteins were described to have overlapped subcellular localization in triple-negative breast carcinoma. Vimentin, an intermediate filament, interacts directly with ERK, activating it, and acts as a scaffold recruiting Slug to ERK promoting Slug phosphorylation and EMT initiation [73].

In addition, *ERK1* was correlated with *ERK2*, and both were correlated with *c-Fos*. It is known that the activation of the transcription factors c-Jun and c-Fos, members of the activating protein-1 (AP-1) family, is induced by growth factors that stimulate ERKs. In the case of c-Fos, the activation of ERK promotes the expression of c-Fos transcript, and ERK-induced c-Fos protein phosphorylation results in its increased transcriptional activity [72].

Finally, it is worth mentioning that the transcriptome of key MAPK/ERK pathway proteins and AQPs, which play important roles in cell proliferation, differentiation, and apoptosis, is closely correlated in pancreatic cancer tissues but not in healthy ones. This suggests a switch in transcriptomics regulation that will impact the physiology of the tissue and influence cancer initiation and progression.

In pancreatic tumor tissues, *AQP5* was correlated with *EGFR*, and *AQP3* and *AQP9* were correlated with *c-Jun*. AQP5 has been shown to activate EGFR and consequently to induce the MAPK signaling pathway in several types of cancer, such as glioma [39], lung adenocarcinoma [53,74], colon cancer [38], and also pancreatic cancer [19]. According to the literature, AQP3 seems to be more associated with FGFR-PI3K and FGFR-ERK signaling pathways, being required for FGF-2-induced cell migration in human breast cancer cells and, thus, influencing cancer metastasis [75]. AQP9 is associated with enhanced androgen-independent prostate cancer progression by activating the ERK pathway and promoting cell proliferation, migration, invasion, and apoptosis [31]. Curiously, in hepatocellular carcinoma, AQP9 seems to promote the inhibition of tumor growth and metastasis via the Wnt/β-catenin pathway, standing as a promising therapeutic target for the treatment of this type of cancer [76].

Previously, we have characterized the implication of AQP3 and AQP5 in ROS accumulation, cell adhesion, proliferation, and motility in human pancreatic cancer BxPC3 cells, further supporting their involvement in pancreatic cancer progression [22,24]. Here, in vitro experiments using BxPC3 cells confirmed the dependence of gene transcriptomics of EMT and MAPK/ERK signaling pathways on *AQP3* and *AQP5* relative expression. As expected, the silencing of *AQP3* and *AQP5* in BxPC3 cells resulted in an impairment of gene expression levels of *CDH1*, *VIM*, *EGFR*, *ERK1*, *ERK2*, *c-Jun*, and *c-Fos*, therefore supporting the Pearson correlation coefficients obtained in human biopsies. The correlation found between AQP expression and these markers’ regulation underscores their potential as putative prognostic biomarkers in pancreatic cancer. Compared to existing biomarkers, such as CA19-9, which lacks specificity and sensitivity, AQP3 and AQP5 offer a mechanistic link to tumor biology, providing insights into disease progression and patient clinical outcomes. Their ability to reflect changes in EMT and MAPK/ERK signaling pathways makes them promising candidates, in conjunction with other established biomarkers, for stratifying patients, predicting therapeutic responses, and identifying high-risk cases.

In vitro experiments using a human pancreatic cancer cell line confirmed that the transcriptional regulation of EMT and MAPK/ERK signaling pathways is influenced by *AQP3* and *AQP5* expression levels. As expected, AQP3 and AQP5 silencing in BxPC3 cells led to a significant reduction in the expression of *VIM*, *EGFR*, *ERK1*, *ERK2*, *c-Jun*, and *c-Fos*, aligning with the Pearson correlation coefficients observed in human biopsy samples. Intriguingly, our data showed a simultaneous reduction of both *CDH1* (E-cadherin) and *VIM* (Vimentin) following *AQP3* knockdown in BxPC3 cells. However, a similar association where AQP3 is positively correlated with increased CDH1 expression was previously reported in wound edges of human chronic wounds [77]. Thus, AQP3 involvement in both epithelial and mesenchymal cellular characteristics cannot be ruled out. These dual correlations imply that, according to specific stimuli, AQP3 could play a role in epithelial–mesenchymal transition (EMT), facilitating dynamic shifts between epithelial adhesion and mesenchymal motility, which are crucial processes in development, wound healing, and cancer progression.

The correlations found ex vivo and in vitro were further validated with publicly available datasets using TNMplot. The observed correlations further support the role of AQP3 and AQP5 in pancreatic cancer progression and reinforce the clinical relevance of targeting AQPs in future therapeutic strategies. These findings strengthen our conclusions and highlight the importance of integrating bioinformatics validation with experimental approaches to gain deeper insights into AQP-mediated signaling mechanisms [78].

Altogether, our results highlight the potential role of AQP3 and AQP5 as prognostic biomarkers in pancreatic cancer. Compared to conventional biomarkers such as CA19-9, which lacks specificity and sensitivity, AQP3 and AQP5 provide a mechanistic link to tumor biology by modulating key oncogenic pathways. Their influence on EMT and MAPK/ERK signaling makes them promising candidates—when combined with established biomarkers—for patient stratification, prognosis prediction, and therapeutic response assessment.

It should be underlined that these results are exploratory and need further investigation with a larger cohort of patients, including the same histological type of pancreatic cancer to avoid sample heterogeneity. Further validation may establish AQP3 and AQP5 as additional prognostic biomarkers for pancreatic cancer, potentially contributing to improving early disease detection, prognosis, and personalized treatment strategies.

## 5. Conclusions

To the best of our knowledge, the present study unveils, for the first time, the interplay between AQPs and key players in EMT and tumorigenesis-related signaling pathways in pancreatic cancer. The strong association found between *AQP5* and *EGFR*, and the associations between *AQP3/AQP9* and *c-Jun* in pancreatic tumor tissues highlight the implications of AQPs in the settings of tumorigenesis. In a nutshell, our study shows that the transcripts of AQPs correlate with the MAPK/ERK signaling pathway in tumor tissues but not in healthy tissues. This finding opens new avenues for further studies focusing on AQPs and their involvement in tumor biology, broadening the biomarker landscape for pancreatic cancer. Additionally, *AQP5* gene expression may be explored as a clinical biomarker for the diagnosis or prognosis of pancreatic cancer.

## Figures and Tables

**Table 1:** Characterization of patients from the pancreatic cancer cohort.

Patient	Pancreatic Cancer Type	Age	Gender	Aggressiveness Grade	Invasiveness Grade
1	Distal cholangiocarcinoma	50	M	Low	High
2	Invasive intraductal papillary mucinous neoplasm	52	M	Low	High
3	Kidney metastasis	55	M	Low	Low
4	Cystic neuroendocrine tumor	56	M	Low	Low
5	Ampulla adenocarcinoma	56	M	Low	High
6	Distal cholangiocarcinoma	58	M	Low	High
7	Ductal adenocarcinoma	59	M	High	High
8	Ampulla adenocarcinoma	63	M	Low	High
9	Ductal adenocarcinoma	64	M	High	High
10	Ductal adenocarcinoma	67	M	High	High
11	Ampulla adenocarcinoma	69	M	Low	High
12	Distal cholangiocarcinoma	69	M	High	High
13	Ductal adenocarcinoma	80	M	Low	High
14	Cystic neuroendocrine tumor	51	F	Low	Low
15	Ductal adenocarcinoma	55	F	High	High
16	Ductal adenocarcinoma	60	F	High	High
17	Ductal adenocarcinoma	65	F	High	High
18	Non-invasive intraductal papillary mucinous neoplasm	65	F	Low	Low
19	Endocrine neoplasia	69	F	High	Low
20	Non-invasive intraductal papillary mucinous neoplasm	69	F	Low	Low
21	Ampulla adenocarcinoma	72	F	Low	High
22	Ductal adenocarcinoma	78	F	High	High
23	Invasive intraductal papillary mucinous neoplasm	80	F	Low	Low
24	Ductal adenocarcinoma	82	F	High	High

M, male; F, female.

**Table 2:** List of qPCR primers.

Gene	Gene Symbol	Primer Sequence
E-cadherin	*CDH1*	FWD: 5′ TCGACACCCGATTCAAAGTG 3′ REV: 5′ GTCCCAGGCGTAGACCAAGA 3′
Vimentin	*VIM*	FWD: 5′ TGCCCTTAAAGGAACCAATGAG 3′ REV: 5′ AGGCGGCCAATAGTGTCTTG 3′
Epidermal growth factor receptor	*EGFR*	FWD: 5′ GAAATCCTCGATGAAGCCTACGTG 3′ REV: 5′ GTCTTTGTGTTCCCGGACATAGTC 3′
Extracellular signal-regulated kinase 1	*ERK1*	FWD: 5′ AAGATCAGCCCCTTCGAACATC 3′ REV: 5′ CTTGTACAGGTCAGTCTCCATCAG 3′
Extracellular signal-regulated kinase 1	*ERK2*	FWD: 5′ TACACCAACCTCTCGTAACATC 3′ REV: 5′ CATGTCTGAAGCGCAGTAAGATT 3′
Jun AP-1 Transcription Factor Subunit	*c-Jun*	FWD: 5′ GTATCCTGCCCAGTGTTGTTTG 3′ REV: 5′ GCAGAAAAGAGGTTAGGGGAGTAC 3′
Fos AP-1 Transcription Factor Subunit	*c-Fos*	FWD: 5′ CCGGGGATAGCCTCTCTTACT 3′ REV: 5′ CCAGGTCCGTGCAGAAGTC 3′
Hypoxanthine-guanine phosphoribosyltransferase 1	*HPRT1*	FWD: 5′ ACTGAACGTCTTGCTCGAGATG 3′ REV: 5′ AGCAGGTCAGCAAAGAATTTATAGC 3′

FWD, forward; REV, reverse.

**Table 3:** Pearson correlation coefficients between *AQP*s, *CDH1*, *VIM*, *EGFR*, *ERK1*, *ERK2*, *c-Jun*, and c-Fos mRNA expression levels in healthy tissues.

					***CDH1***	***VIM***	***EGFR***	***ERK1***	***ERK2***	***c-Jun***	***c-Fos***
				***AQP1***	−0.254 0.310	−0.116 0.648	−0.255 0.307	−0.289 0.244	−0.142 0.588	−0.257 0.273	−0.361 0.141
				***AQP3***	−0.195 0.469	0.038 0.890	−0.196 0.468	−0.094 0.730	−0.008 0.663	−0.108 0.671	−0.213 0.413
				***AQP5***	−0.325 0.477	−0.294 0.480	−0.293 0.444	−0.332 0.422	−0.203 0.601	0.169 0.664	−0.423 0.344
	***AQP1***	***AQP3***	***AQP5***	***AQP9***	−0.312 0.452	−0.036 0.932	−0.237 0.511	−0.143 0.694	−0.258 0.473	−0.184 0.611	−0.266 0.489
***CDH1***	−0.254 0.310	−0.195 0.469	−0.325 0.477	−0.312 0.452		0.487 **0.041**	0.397 0.115	0.211 0.401	0.400 0.111	−0.144 0.557	0.892 **<0.001**
***VIM***	−0.116 0.648	0.038 0.890	−0.294 0.480	−0.036 0.932	0.487 **0.041**		0.466 0.059	0.505 **0.033**	0.707 **0.002**	−0.177 0.470	0.742 **<0.001**
***EGFR***	−0.255 0.307	−0.196 0.468	−0.293 0.444	−0.237 0.511	0.397 0.115	0.466 0.059		0.135 0.592	0.058 0.826	0.397 0.103	0.617 **0.008**
***ERK1***	−0.289 0.244	−0.094 0.730	−0.332 0.422	−0.143 0.694	0.211 0.401	0.505 **0.033**	0.135 0.592		0.842 **<0.001**	0.002 0.994	0.607 **0.008**
***ERK2***	−0.142 0.588	−0.008 0.663	−0.203 0.601	−0.258 0.473	0.400 0.111	0.707 **0.002**	0.058 0.826	0.842 **<0.001**		−0.137 0.587	0.567 **0.022**
***c-Jun***	−0.257 0.273	−0.108 0.671	0.169 0.664	−0.184 0.611	−0.144 0.557	−0.177 0.470	0.397 0.103	0.002 0.994	−0.137 0.587		0.020 0.937
***c-Fos***	−0.361 0.141	−0.213 0.413	−0.423 0.344	−0.266 0.489	0.892 **<0.001**	0.742 **<0.001**	0.617 **0.008**	0.607 **0.008**	0.567 **0.022**	0.020 0.937	

In each cell, the upper number represents the Pearson correlation coefficient (r) value, and the lower number represents the exact *p*-value found for each correlation. *p* < 0.05 values are shown in bold.

**Table 4:** Pearson correlation coefficients between *AQP*s, *CDH1*, *VIM*, *EGFR*, *ERK1*, *ERK2*, *c-Jun*, and *c-Fos* mRNA expression levels in pancreatic tumor tissues.

					***CDH1***	***VIM***	***EGFR***	***ERK1***	***ERK2***	***c-Jun***	***c-Fos***
				***AQP1***	−0.235 0.319	−0.051 0.850	−0.145 0.520	−0.238 0.357	−0.255 0.266	0.282 0.228	−0.239 0.311
				***AQP3***	−0.210 0.361	−0.287 0.265	−0.128 0.562	−0.202 0.421	−0.212 0.343	0.852 **<0.001**	−0.083 0.721
				***AQP5***	0.737 **<0.001**	−0.064 0.827	0.457 **0.043**	−0.334 0.223	0.148 0.546	0.158 0.532	−0.121 0.631
	***AQP1***	***AQP3***	***AQP5***	***AQP9***	−0.124 0.625	−0.272 0.347	−0.092 0.698	−0.185 0.508	−0.144 0.545	0.952 **<0.001**	−0.049 0.848
***CDH1***	−0.235 0.319	−0.210 0.361	0.737 **<0.001**	−0.124 0.625		0.441 0.100	0.744 **<0.001**	0.689 **0.001**	0.395 0.076	−0.320 0.169	0.063 0.787
***VIM***	−0.051 0.850	−0.287 0.265	−0.064 0.827	−0.272 0.347	0.441 0.100		−0.073 0.781	0.719 **0.006**	0.361 0.170	−0.237 0.359	0.066 0.808
***EGFR***	−0.145 0.520	−0.128 0.562	0.457 **0.043**	−0.092 0.698	0.744 **<0.001**	−0.073 0.781		0.259 0.285	0.098 0.658	−0.239 0.284	0.150 0.506
***ERK1***	−0.238 0.357	−0.202 0.421	−0.334 0.223	−0.185 0.508	0.689 **0.001**	0.719 **0.006**	0.259 0.285		0.796 **<0.001**	−0.192 0.445	0.567 **0.014**
***ERK2***	−0.255 0.266	−0.212 0.343	0.148 0.546	−0.144 0.545	0.395 0.076	0.361 0.170	0.098 0.658	0.796 **<0.001**		−0.217 0.345	0.667 **0.001**
***c-Jun***	0.282 0.228	0.852 **<0.001**	0.158 0.532	0.952 **<0.001**	−0.320 0.169	−0.237 0.359	−0.239 0.284	−0.192 0.445	−0.217 0.345		−0.153 0.508
***c-Fos***	−0.239 0.311	−0.083 0.721	−0.121 0.631	−0.049 0.848	0.063 0.787	0.066 0.808	0.150 0.506	0.567 **0.014**	0.667 **0.001**	−0.153 0.508	

In each cell, the upper number represents the Pearson correlation coefficient (r) value, and the lower number represents the exact *p*-value found for each correlation. *p* < 0.05 values are shown in bold.

## Data Availability

The raw data underlying the present study are accessible to readers upon request.
